# MODY screening: a new center for molecular genetic diagnosis in Brazil

**DOI:** 10.1186/1758-5996-7-S1-A213

**Published:** 2015-11-11

**Authors:** Lucas Santos de Santana, Lílian Araújo Caetano, Márcia Nery, Alexander Augusto de Lima Jorge, Milena Gurgel Teles Bezerra

**Affiliations:** 1 FMUSP, São Paulo, Brazil

## Background

Maturity-Onset Diabetes of the Young (MODY) is a form of monogenic diabetes characterized by autosomal dominant inheritance, young age of onset and pancreatic beta-cell dysfunction without autoimmune cause. To date 13 genes have been identified associated with MODY phenotype, with four of them (HNF1A; GCK; HNF4A and HNF1B) being responsible for over 95% of cases. Recently, our group has initiated molecular genetic screening for MODY using Sanger's sequencing method.

## Objective

To report novel mutations related to MODY 2 (GCK) and 3 (HNF1A) of a large cohort of Brazilian diabetic families in a new MODY diagnostic center.

## Materials and methods

A total of 126 subjects from 63 Brazilian families were screened for GCK or HNF1A mutations: 30 families with clinical suspicion of MODY 2 and 33 families with MODY 3 phenotype. All exons and adjacent intronic regions of GCK and HNF1A genes were studied.

## Results

We found 16 GCK mutations (11 nonsynonymous, 2 splice site and 3 frameshift-deletion) in 30 families (53%). Five of these variants were novel: c.580-3C>A (IVS5); c.505A>G/p.K169E; c.110T>C/p.M37T; c.326_326delC/p.I110Sfs*6 and c.1247_1247delA/p.H416Pfs*15. HNF1A mutations were found in 6 families (18%): 2 nonsynonymous, 1 nonsense and 3 frameshift-insertion. One was a novel mutation: c.1558C>T/p.Q520*. All 6 novel variants were absent in databases of healthy controls (1KG/ESP-6500) and were predicted to be damaging using in silico analysis (Polyphen-2, MutationTaster, SIFT/PROVEAN, Human Splicing Finder). In variants p.K169E and p.M37T, the substitution occurred in a codon already associated with MODY. All 6 subjects with novel variants have diabetes onset before age of 25 and BMI below 25. Five of them have family history of diabetes and one have parents without impaired fasting glucose. Familial co-segregation analysis was possible in 3 of the 6 probands, in which the variant segregated in family members with diabetes. All of them had detectable fasting C-peptide (range 0,7-2,3 ng/mL) 3 yrs. after diagnosis of diabetes, and negative beta cell pancreatic antibodies (GAD, IA2, IAA).

## Conclusion

This molecular study has identified 6 unpublished mutations in subjects with clinical features of MODY, expanding the number of variants associated to this phenotype.

